# Identifying USPs regulating immune signals in *Drosophila*: USP2 deubiquitinates Imd and promotes its degradation by interacting with the proteasome

**DOI:** 10.1186/s12964-014-0041-2

**Published:** 2014-07-16

**Authors:** Elodie Engel, Perrine Viargues, Magda Mortier, Emmanuel Taillebourg, Yohann Couté, Dominique Thevenon, Marie-Odile Fauvarque

**Affiliations:** 1 Univ.Grenoble Alpes, iRTSV, BGE, Grenoble, F-38000, France; 2 CEA, DSV, iRTSV, BGE, Grenoble, F-38000, France; 3 INSERM, BGE, U1038, Grenoble, F-38000, France; 4 iRTSV, BGE, CEA-Grenoble, 17 rue des Martyrs, Grenoble Cedex, 38054, France

**Keywords:** Innate immunity, Imd, NF-κB, Proteasome, RNA interference screen, Toll, Ubiquitin specific protease, USP2 (CG14619), USP34 (CG5794), USP36 (CG5505)

## Abstract

**Background:**

Rapid activation of innate immune defences upon microbial infection depends on the evolutionary conserved NF-κB dependent signals which deregulation is frequently associated with chronic inflammation and oncogenesis. These signals are tightly regulated by the linkage of different kinds of ubiquitin moieties on proteins that modify either their activity or their stability. To investigate how ubiquitin specific proteases (USPs) orchestrate immune signal regulation, we created and screened a focused RNA interference library on *Drosophila* NF-κB-like pathways Toll and Imd in cultured S2 cells, and further analysed the function of selected genes *in vivo.*

**Results:**

We report here that USP2 and USP34/Puf, in addition to the previously described USP36/Scny, prevent inappropriate activation of Imd-dependent immune signal in unchallenged conditions. Moreover, USP34 is also necessary to prevent constitutive activation of the Toll pathway. However, while USP2 also prevents excessive Imd-dependent signalling *in vivo*, USP34 shows differential requirement depending on NF-κB target genes, in response to fly infection by either Gram-positive or Gram-negative bacteria. We further show that USP2 prevents the constitutive activation of signalling by promoting Imd proteasomal degradation. Indeed, the homeostasis of the Imd scaffolding molecule is tightly regulated by the linkage of lysine 48-linked ubiquitin chains (K48) acting as a tag for its proteasomal degradation. This process is necessary to prevent constitutive activation of Imd pathway *in vivo* and is inhibited in response to infection. The control of Imd homeostasis by USP2 is associated with the hydrolysis of Imd linked K48-ubiquitin chains and the synergistic binding of USP2 and Imd to the proteasome, as evidenced by both mass-spectrometry analysis of USP2 partners and by co-immunoprecipitation experiments.

**Conclusion:**

Our work identified one known (USP36) and two new (USP2, USP34) ubiquitin specific proteases regulating Imd or Toll dependent immune signalling in *Drosophila*. It further highlights the ubiquitin dependent control of Imd homeostasis and shows a new activity for USP2 at the proteasome allowing for Imd degradation. This study provides original information for the better understanding of the strong implication of USP2 in pathological processes in humans, including cancerogenesis.

## Lay abstract

Inflammation is a major player of our innate immune defences which is induced within minutes following infection by microorganisms. Its activation mainly depends on intracellular signals inducing the secretion of pro-inflammatory cytokines and anti-microbial molecules. This high reactivity implies mechanisms preventing inappropriate activation of pro-inflammatory immune signals that can otherwise favours cancer progression or induce tissue damages such as those occurring in auto-immune diseases. We used *Drosophila* flies model system for the identification of new enzymes of the ubiquitin specific protease family regulating evolutionary conserved immune signals in both *Drosophila* and Humans. These enzymes specifically control the stability or the activated status of target proteins by hydrolysing a small peptide, called ubiquitin, which is linked as a monomer or polymers on protein. Moreover, they constitute promising and yet poorly explored targets for the finding of drugs useable as therapeutic molecules. We found a set of three ubiquitin specific proteases regulating immune signals in response to infections, among which USP2 controls the homeostasis of an essential signalling component -named Imd- by promoting its degradation at the proteasome. Beyond the immune response, this work highlights how a specific enzyme, USP2, may regulate its targets in physio-pathological processes for a better understanding of its pathogenic activity in cancers and inflammation.

## Background

Conjugation of ubiquitin monomers or polymers to proteins is a key mechanism for controlling their activity or stability [[1]]. Lysine (Lys) residues of proteins can be modified by a single ubiquitin monomer or by polymers of ubiquitin (polyubiquitin) each linked through Lys 48 (K48) or through Lys63 (K63) of the ubiquitin molecule, or by other kinds of polymers. Whereas K48-linked polyubiquitin (Ub^K48^) mainly triggers degradation of proteins by the proteasome, monoubiquitination, K63-linked polyubiquitin (Ub^K63^) and other polyubiquitin chains regulate the activity, the conformation or the subcellular localisation of proteins [[2],[3]]. Mammalian genomes contain about one hundred ubiquitin proteases – the enzymes that remove ubiquitin moieties from proteins- that are divided in five subfamilies, among which the Ubiquitin Specific Proteases (USPs) subfamily represents the major class in both human and *Drosophila* [[4]-[7]]. The NF-κB dependent signalling pathways, that are central to pro-inflammatory and immune signalling, are tightly regulated by the ubiquitination of several of their protein components including RIP1, interacting with the tumour necrosis factor receptor 1 (TNF-R1), and downstream protein kinase complexes [[5],[8]]. A growing number of ubiquitin proteases have been found to mediate transient inhibition of NF-κB- pathways frequently sharing a same target which raises the question of their specificity and functional interrelationships [[5]].

In *Drosophila*, two conserved NF-κB-like signalling pathways, Toll and Imd, contribute to innate immunity by promoting the expression of antimicrobial peptide (AMP) encoding genes -mainly in fat body cells- in response to infections [[9]]. The Toll receptor is activated by binding of the processed cytokine Spätzle (Spz), whose cleavage depends on upstream extracellular proteolytic cascades initiated by circulating peptidoglycan recognition proteins (PGRPs) following the recognition of various pathogens associated patterns, such as the Lys-type peptidoglycans from Gram-positive bacteria [[10],[11]]. Activated Toll associates with adaptor proteins dMyd88 and Tube, ultimately resulting in the activation of the NF-κB like factors, Dif and/or Dorsal (DL), which activate the transcription of a set of AMP encoding genes including *Drosomycin* (*Drs*), immune induced molecule 1 (*IM1)* and *Attacin* (*AttA*) [[10],[12]-[14]]. The Imd pathway is induced by the direct binding of diaminopimelic acids containing bacterial peptidoglycan fragments to the transmembrane receptor PGRP-LC/Ird7 and results in the activation of another set of AMPs encoding genes, including *Diptericin* (*Dpt*), *Defensine* (*Def*) and *Attacin A* (*AttA)* [[12]]. PGRP-LC associates with the cytoplasmic scaffolding protein Imd [[15]] which mediates the activation of Tak1 and Kenny (Key), the *Drosophila* IKKγ homolog, resulting in the phosphorylation of the NF-κB like factor Relish (Rel) [[16]].

We have previously demonstrated that Imd is ubiquitinated by Ub^K63^ and that the ubiquitin specific protease USP36/Scny negatively regulates signal transduction by hydrolysing Ub^K63^ from Imd [[17]]. Complex regulation of immune signals by ubiquitin-dependent mechanisms prompted us to identify other ubiquitin specific proteases (USPs) acting in Imd and Toll dependent immune signalling. To this end, we have designed and screened an RNAi library targeting the *Drosophila* USPs in S2 cells. We report here the identification of three regulators of the Imd pathway: the already described USP36 and two new USPs, USP2 and USP34/Puf (Puffyeye [[18]]), and of one regulator of the Toll pathway: USP34. We demonstrate that USP2 and USP34 are required to prevent constitutive activation of immune signalling *in vivo*. However, while USP2 is also required to prevent excessive activation of the Imd pathway in infected flies, USP34 displays differential requirement depending on the antimicrobial peptide gene analysed: silencing *Usp34* enhanced the activation of *AttA*, *Drs* and *IM1*. In the opposite, USP34 is required for full activation of the two specific Imd-dependent genes *Dpt* and *Def* in response to Gram-negative bacteria. These results suggest a complex requirement of USP34 in these two pathways. Focusing on USP2 biochemical function, we show that USP2 binds to Imd and promotes the cleavage of Ub^K48^ chains from the protein in both cultured cells and flies. Surprisingly, USP2 also targets Imd for degradation, a function which, from our proteomic analysis of USP2 partners and subsequent co-immunoprecipitation experiments, likely occurs at the level of the proteasome. Interestingly, Ub^K48^ chains linkage on Imd, acting as a tag for its proteasomal degradation, is fully prevented in response to infection therefore ensuring the required protein stabilisation for signal transduction. Thus, Imd homeostasis is tightly regulated by ubiquitination and USP2 controls Imd pathway activation by regulating the steady-state level of the Imd protein.

## Results

### Three USPs regulate the Imd pathway in S2 cells

**Figure 1:**
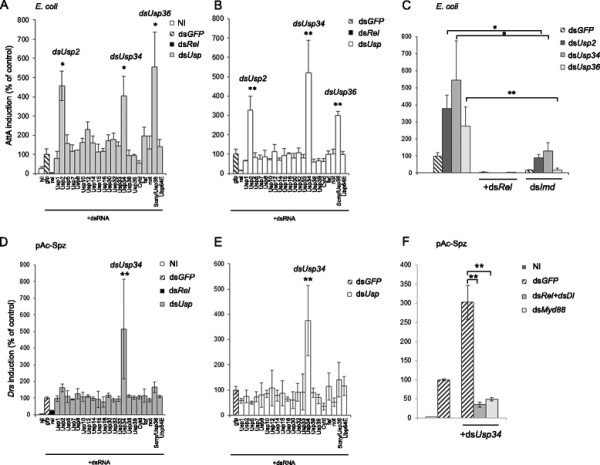
**Screening USPs regulating Imd and Toll pathways.***Drosophila* S2 cells were co-transfected with the indicated dsRNAs, *pAct-luc* normaliser and either the *pAttA-luc***(A-C)** or the *pDrs-luc***(D-F)** reporter gene. **A**,**C**: The Imd pathway was activated by adding heat-killed *E. coli*. **D**,**F**: The Toll pathway is stimulated by co-transfecting cells with *pAc-Spz*. **B,E**: Cells are not stimulated (ie, neither by *E. coli***(B)** not by pAc-Spz **(E)**) in order to look after constitutive deregulation of the pathway. **A**,**D**,**C**,**F**. NI: not induced control. **A**-**F**. Histograms represent the % of induction compared to double strand *gfp* treated control cells. One out of three independent experiments is shown. Error bars indicate standard deviation of technical triplicates. Significant differences (Student-*t*-test) p < 0.05 (*), p < 0.02 (**).

We created a double strand RNA interference (dsRNA) library targeting USPs encoded in the *Drosophila* genome (Additional file 1: Table S1) (see also [[7],[19]]). This library was screened in *Drosophila* S2 cells on PGRP-LC-dependent activation of Imd pathway mediated by adding heat-killed *Escherichia coli (E. coli)* in the culture medium. Activation of the pathway was monitored by using an *Attacin*-*luciferase* (*AttA-luc*) reporter gene and the signal was normalised to that from a control *Actin-luciferase* (*Act-luc*) reporter gene [[20]]. Silencing *Usp2*, *Usp34* and *Usp36* genes resulted in over-activation of the Imd dependent *AttA* promoter whereas silencing the other *Usp* genes had no significant effect (Figure 1A). Constitutive activation of the *AttA* promoter was also detected when the same three *Usp* genes were silenced in the absence of heat-killed *E. coli* (Figure 1B). Monitoring *Usp2, Usp34* and *Usp36* transcripts in RNAi-treated cells showed effective gene silencing (Additional file 2: Figure S1). Activation of the Imd pathway in *Usp2*, *Usp34* and *Usp36* silenced cells was strictly dependent on the NF-κB like protein Rel since co-silencing *Rel* with each of these three genes fully prevented activation of the *AttA* promoter (Figure 1C). Co-silencing *Imd* with either *Usp2* or *Usp34* strongly decreased the induction observed in only *Usp2*- or *Usp34*-silenced cells indicating a crucial contribution of Imd (Figure 1C). Finally, as expected from our previous findings demonstrating that USP36 directly targets Imd [[17]], we observed no induction of *AttA* when *Imd* and *Usp36* were co-silenced in S2 cells (Figure 1C). This screen thus defines USP2, USP34 and USP36 as inhibitors of the Imd pathway in S2 cells.

### USP34 inhibits the Toll pathway in S2 cells

To identify ubiquitin proteases regulating the Toll pathway, we activated this pathway by expressing Spz -the ligand of Toll- in S2 cells. Expression of a *Drs-luc* reporter construct was monitored and normalized to that of an *Act-luc* construct [[21]]. A preliminary assay indicated that the NF-κB factors Dorsal (Dl) and Rel, but not Dif, were required for the Toll-mediated activation of the *Drs* promoter in S2 cells (Additional file 2: Figure S2) as also described in a previous study [[22]]. Silencing upstream components of the pathway, *dMyd88* or *Spz*, prevented the activation of the *Drs* promoter as expected (Additional file 2: Figure S2). By contrast, silencing upstream components of the Imd pathway, *PGRP-LC, Imd, Tab2* and *Key,* did not affect expression from the *Drs* promoter induced by Spz, indicating that Rel is the sole Imd pathway component contributing to Toll activation in S2 cells (Additional file 2: Figure S2). Screening the RNAi library in Spz-expressing cells revealed that *Usp34* silencing resulted in a significant enhancement of *Drs* promoter activity when compared to control cells (Figure 1D). Similarly, *Usp34* was the only gene whose silencing induced the *Drs* promoter in cells not expressing Spz (Figure 1E). This activation was strictly dependent on downstream NF-κB factors (Rel and Dl) and on the adaptor protein dMyd88 (Figure 1F). We therefore identified USP34 as a new inhibitor of the Toll pathway both in stimulated and un-stimulated cells.

### Overexpressing USP2 or USP34 suppresses fly immunity *in vivo*

**Figure 2:**
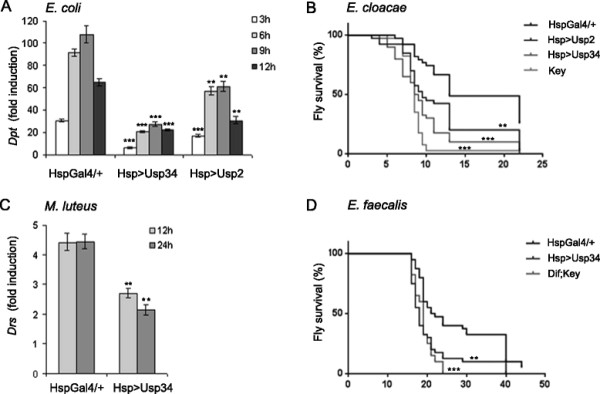
**Overexpressing USP2 or USP34 suppresses fly immunity*in vivo.*A**-**D**. Flies were heat shocked 12 hours before infection. **A**. Quantitative analysis of *Dpt* mRNAs levels by RT-qPCR at 3, 6, 9 and 12 hours post infection with *E. coli*. **B**. Forty heat-shocked flies of 5 days old were infected with *E. cloacae* and their survival kinetics was followed over 24 hours. The mutant *key* is used as a Gram-negative bacteria sensitive control (non-heat shocked flies). **C**. Quantitative analysis of *Drs* mRNAs by RT-qPCR at 12 and 24 hours post infection with *M. luteus*. **D**. Forty flies of 5 days old were infected with *E. faecalis* 6 hours following heat shock treatment and their survival kinetics was followed over 48 hours. The mutant *w*^*1118*^*;Dif,Key* is used as a Gram-positive bacteria sensitive control (non-heat shocked flies). **A**,**C**: Results are expressed as the fold induction level compared to the level of *Dpt***(A)** or *Drs***(C)** mRNAs in non-infected flies. Error bars indicate standard deviation between technical replicates. One representative experiment out of three is shown. Significant difference: p < 0.05 (*), p < 0.002 (**) or p < 0.0001 (***) compared to HspGal4/+control flies (Student’s-*t*-test). **B**,**D**: Results are expressed as % of surviving flies. One representative experiment out of three is shown. Significant difference: p < 0.002 (**) or p < 0.0001 (***) compared to HspGal4/+control flies (Log-rank (Mantel-Cox) test). **A**-**D**. Flies’ genotypes are: *w*^*1118*^;HspGal4/+ (HspGal4/+), *w*^*1118*^;HspGal4/+; P{EPgy2}ash2EY03971/+ (Hsp > Usp34), *w*^*1118*^;HspGal4/+;UAS-*Usp2/+* (Hsp > Usp2), *w*^*1118*^*;key*^*1*^ (*key*) and *w*^*1118*^*; Dif*^*1*^*, key*^*1*^(*Dif,key).*

The USP36 negative function on flies resistance to infection and antimicrobial peptide expression has been previously described [[17]]. To assess the function of USP2 and USP34 on immune signalling *in vivo*, we created a UAS-*Usp2* transgenic strain and used a P[UAS] insertion located upstream of the *Usp34* transcription unit, P[EPgy2]ash2^EY03971^ (hereafter referred to as UAS-*Usp34*), in order to overexpress each corresponding genes under the control of the GAL4 transcription factor [[23]]. Overexpression of these two genes was induced in adults through the heat-shock HspGal4 driver line and was verified by quantitative Real Time PCR at 3, 6, 9 and 12 hours post-heat shock (Additional file 2: Figure S3). A strong diminution of *Dpt* expression was observed in *E. coli* infected flies overexpressing USP34 and to a lesser but significant extent, in flies overexpressing USP2 (Figure 2A). Inhibition of AMP gene expression was associated with increased fly sensitivity to the Gram-negative pathogen *Enterobacter cloacae* (Figure 2B) and *Klebsellia pneumonia* (not shown)*.* Similarly as observed for *Dpt*, overexpressing USP34 significantly reduced *Drs* expression at 12 and 24 hours after infection with the non-pathogenic Gram-positive bacteria *Micrococcus luteus* (Figure 2C). This phenotype was associated with increased fly sensitivity to the Gram-positive pathogen *Enterococcus faecalis* (Figure 2D). Therefore, when overexpressed *in vivo,* USP2 and USP34 behave as negative regulators of the fly immune response to bacterial infections.

### USP2 and USP34 prevent constitutive immune signalling *in vivo* but differentially control antimicrobial peptide genes expression following bacterial infection

**Figure 3:**
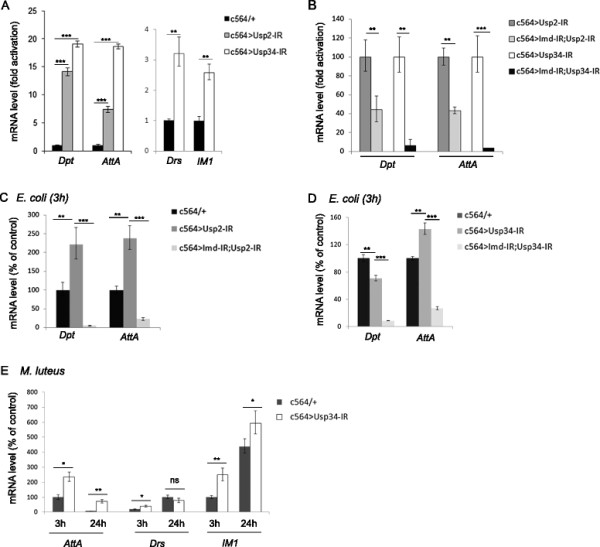
**USP2 and USP34 regulate immune signalling*in vivo*.** Quantitative analysis of *Dpt*, *AttA*, *Drs and IM1* mRNAs by RT-qPCR. **A**. Histograms present the fold activation of the indicated gene mRNA in *Usp2* (c564 > Usp2-IR) or *Usp34* (c564 > Usp34-IR) silenced flies compared to control flies (c564Gal4/+) raised in similar conditions. **B**. *Dpt* and *AttA* induction in double silenced flies (c564 > Usp2-IR, Imd-IR or c564 > Usp34-IR, Imd-IR) expressed as a % of activity of *Usp*-only silenced flies (c564 > Usp2-IR or c564 > Usp34-IR, respectively). **C**,**D**. Activation of *Dpt* and *AttA* in *Usp2*- **(C)** or *Usp34***(D)** -silenced flies, in combination or not with an *Imd*-silencing transgene-, at 3 hours post *E. coli* infection, expressed as a % of activity compared to infected control flies (c564/+). **E**. Activation of *AttA, Drs* and *IM1* in *Usp34*-silenced flies at 3 and 24 hours post *M. luteus* infection expressed as a % of activity compared to infected control flies (c564/+) (100% is the maximal activation observed in control flies at 3 hours post infection for *AttA*, and *IM1* and at 24 hours post-infection for *Drs*). **A**-**E**. One representative experiment out of three is shown. Error bars indicate standard deviation between technical triplicates. Significant differences (*t*-test), p < 0.05 (*), p < 0.02 (**), p < 0.001 (***).

To investigate further the functions of USP2 and USP34 *in vivo*, we created transgenic fly lines expressing inverted repeats (IR) of *Usp2* (Usp2-IR #5M hereafter designed as Usp2-IR) or *Usp34* (Usp34-IR #1M hereafter designed as Usp34-IR) inducing efficient gene silencing in the living flies (Additional file 2: Figure S4A,B)*.* Specific silencing of either *Usp2* or *Usp34* in the adult fat body was then achieved by using the driver line *c564* [[24]] and resulted in a significant constitutive activation of *Dpt* and *AttA* in the absence of immune challenge (Figure 3A). We obtained similar results with other transgenes carrying different silencing sequences designed to silence the same two genes from the VDRC collection [[25]] (Additional file 2: Figure S4A-D). In addition, a significant three-fold activation of two Toll-pathway target genes *Drs* and *IM1* was also observed in *Usp34-*silenced flies (Figure 3A). Silencing *Imd* strongly reduced the constitutive activation of Imd-dependent target genes *AttA* and *Dpt* induced by silencing either *Usp2* or *Usp34* indicating that Imd contribute to the observed up-regulation of these antimicrobial genes (Figure 3B). Moreover, enhanced activation of *Dpt* and *AttA* expression was observed in *Usp2*-silenced flies three hours after a septic injury with *E. coli* and this activation was fully prevented in *Imd*-silenced flies (Figure 3C). Enhanced activation of *Dpt* and *AttA* in *Usp2*-silenced flies was similarly observed at several time-points from 3 to 9 hours following infection (Additional file 2: Figure S5A).

In contrast, antimicrobial peptide gene induction upon bacterial infection was differentially modified in *Usp34*-silenced flies. Indeed, in flies infected by *E. coli*, the induction of *AttA* was enhanced in *Usp34* silenced flies compared to control flies (Figure 3D, Additional file 2: Figure S5C). However, the induction of *Dpt* expression was significantly compromised in *Usp34*-silenced flies, resulting in a 30 to 50% reduction of *Dpt* induction from 3 to 9 hours following infection by *E. coli* (Figure 3D, Additional file 2: Figure S5C). The expression of *Defensin C (Def)* an additional Imd-dependent antimicrobial peptide encoding gene, was also significantly deregulated in both *Usp34*-silenced non-infected flies and at 3 hours post-infection (Additional file 2: Figure S5B,C) but then, it was strongly compromised from 6 to 9 hours following *E. coli* infection (Additional file 2: Figure S5C). Finally, when the Toll pathway was specifically activated by infecting flies with *M. luteus*, all three target genes analyzed, *AttA*, *Drs* and *IM1* were significantly enhanced in *Usp34* silenced flies compared to control flies at 3 hours following the infection, while at 24 hours infection, up-regulation was significant only in the cases of *AttA* and *IM1* (Figure 3E).

These data suggest that in non-infected flies, both USP2 and USP34 are required in fat body cells to inhibit immune signals. However, in infected flies, USP2 is acting as a negative regulator but USP34 is differentially required for either the activation or the inhibition of antimicrobial peptide genes.

Finally, the monitoring of *Usp2* and *Usp34* mRNAs in response to *E. coli* or *M. luteus* infections revealed no major change in *Usp2* or *Usp34* gene expression (below 1,6 fold change) (Additional file 2: Figure S6). This suggests that the activity or stability of these two proteases in response to infection is mainly regulated at the protein level.

### USP2 interacts with Imd and promotes cleavage of Imd-linked Ub^K48^

**Figure 4:**
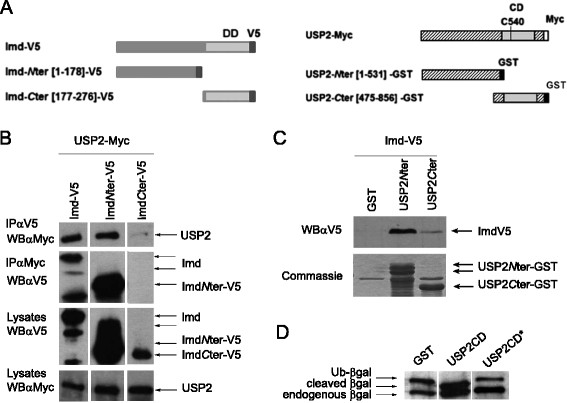
**USP2 interacts with Imd. A**. Representation of the constructs used in this study. DD: death domain in Imd, CD: catalytic domain in USP2, V5: V5 tag, Myc: Myc tag. Numbers in brackets indicate amino-acid position. **B**. *Drosophila* S2 cells were cotransfected with the expression constructs encoding USP2-Myc and either Imd-V5, or Imd*-N-*ter-V5 or Imd*-C-*ter-V5 as indicated. Cells lysates were coimmunoprecipitated (IP) with either anti-V5 or anti-Myc antibodies and analysed by western blot (WB) with either anti-Myc or anti-V5 antibodies as indicated. **C**. S2 cells were transfected with the expression construct encoding Imd-V5 and lysed after 48 h. Cells lysates were pre-cleared and subjected to GST pull down assays using GST fusion proteins with either the USP2-*N*-ter [1–531] or the USP2-*C*-ter [475–856] - or with GST alone (GST). Gel was coloured with Coomassie to visualize GST-fusion proteins in the input (bottom part). **D**. GST-fusion of the wild type (USP2CD) or mutated (USP2CDC540S indicated USP2CD*) catalytic domain of USP2 were coexpressed with Ub-β-gal for 4 hours at 28°C in transformed *E. coli* XL1 Blue. Substrate cleavage was analysed by western blotting with anti-βgal antibodies.

We decided to focus on the function of USP2 in the Imd pathway. So, we transfected USP2-Myc and Imd-V5 tagged expressing constructs in S2 cells to assess whether the corresponding proteins interact with each other (Figure 4A). Indeed, we observed co-immunoprecipitation of the two proteins indicating that USP2 and Imd belong to a same protein complex (Figure 4B). Imd is a scaffolding protein containing a death domain located in the *C*-terminus part of the protein while its *N*-terminus part is required for its interaction with PGRP-LC [[15],[26]]. Using V5 tagged Imd truncated constructs (Figure 4A), we showed that USP2 preferentially interacts with the PGRP-LC-interacting Imd-*N-*ter part in S2 cells (Figure 4B). GST pull-down assays using GST-tagged full-length and truncated forms of Imd expressed as recombinant proteins in bacteria confirmed this preferential interaction (Additional file 2: Figure S7).

The catalytic domain of USP2 - containing the critical cysteine residue required for ubiquitin chain hydrolysis at position 540- is located in the *C-*terminal part of the enzyme [[27]] (Figure 4A). By using GST pull-down assays with GST-tagged full length or truncated forms of USP2 (USP2*-N-*ter [AA:1–531] and USP2*-C-*ter [AA:475–856]), we finally showed that USP2 preferentially interacts with Imd through its non-catalytic *N*-ter domain (Figure 4C).

We then expressed the USP2 *-C-*ter catalytic domain (USP2^CD^) in bacteria expressing a fusion protein of ubiquitin with β-galactosidase (Ub-β-gal), a substrate for deubiquitination. We observed a clear cleavage of this substrate indicating that *Drosophila* USP2 is an active deubiquitinating enzyme (Figure 4D). Mutation of the conserved Cys540 to Ser (C540S) resulted in no hydrolysis of the Ub-β-gal fusion protein (Figure 4D, USP2^CD*^).

**Figure 5:**
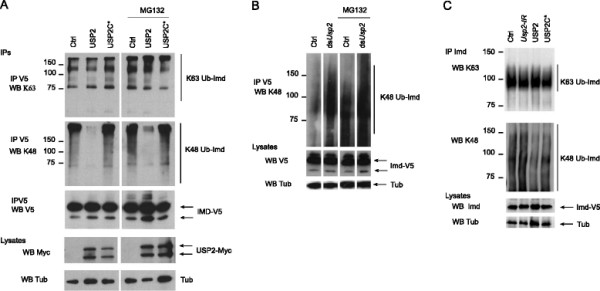
**USP2 hydrolyses Imd-linked Ub**^**K48**^**. A**. *Drosophila* S2 cells were cotransfected with Imd-V5 and either wild type USP2 or mutant USP2C540S (USP2C*) expressing constructs in untreated cells or cells treated with MG132 at 20 μM for 4 hours before cell lysis. Imd-V5 was immunoprecipitated with anti-V5 antibodies (IP V5) and Imd-ubiquitinated forms were detected by western blot with either anti-Ub^K63^ (WB K63) or anti-Ub^K48^ (WB K48) antibodies as indicated. Imd full length was detected with anti-V5 antibodies (IP V5 WB V5). Expression of USP2 was assessed by western blot of whole cell lysate with anti-Myc antibodies (WB Myc). **B**. *Drosophila* S2 cells were cotransfected with Imd-V5 in the presence or not of *Usp2* silencing dsRNA (ds*Usp2*). Imd-V5 was immunoprecipitated with anti-V5 antibodies and Imd-ubiquitinated forms were detected by western blot with anti-Ub^K48^ antibodies (IP V5 WB K48). Imd full length was detected in the cell lysate with anti-V5 antibodies (WB V5). **C**. Endogenous Imd was immunoprecipitated with anti-Imd antibodies from extracts of c564/+ control flies (Ctrl), or flies expressing either the *Usp2* silencing transgene (*Usp2*-IR), or the wild type (USP2) or the catalytically inactive form (USP2C*) of USP2, under the control of the c564Gal4 driver line (IP Imd). Ubiquitinated forms of Imd were detected with either Ub^K63^ (WB K63) or Ub^K48^ (WB K48) antibodies as indicated. The amount of endogenous Imd proteins was detected in total fly extracts with Imd antibodies (WB Imd). **A**-**C**. Anti-tubulin antibodies served as loading control (WB Tub).

To test the ability of USP2 to deubiquitinate Imd, we co-expressed full-length USP2 with Imd in S2 cells. Whereas the level of Ub^K63^-Imd was not modified by the presence of wild type or catalytically inactive full length USP2, the amount of Ub^K48^-Imd was reduced in cells overexpressing USP2 but not in cells expressing its catalytically inactive form in both untreated or MG132 treated cells (Figure 5A). This suggests that USP2 specifically hydrolyses Imd-linked Ub^K48^. Consistent with this hypothesis, Ub^K48^-Imd accumulated in *Usp2*-silenced cells both in MG132 treated or untreated cells (Figure 5B).

To characterize the activity of USP2 on Imd-linked ubiquitin chains *in vivo*, we immunoprecipitated the endogenous Imd protein from flies extracts. Silencing *Usp2* resulted in the accumulation of Ub^K48^-Imd (Figure 5C). On the opposite, expressing USP2, but not the catalytic mutant, resulted in a strong diminution of immunoprecipitated Ub^K48^-Imd compared to control flies (Figure 5C). As observed in S2 cells, Ub^K63^-Imd chains were not affected by *Usp2* gene extinction or overexpression (Figure 5C). Therefore, we conclude that *Drosophila* USP2 is an active deubiquitinating enzyme specifically promoting the hydrolysis of Ub^K48^ linked to Imd.

### USP2 promotes Imd degradation by the proteasome

**Figure 6:**
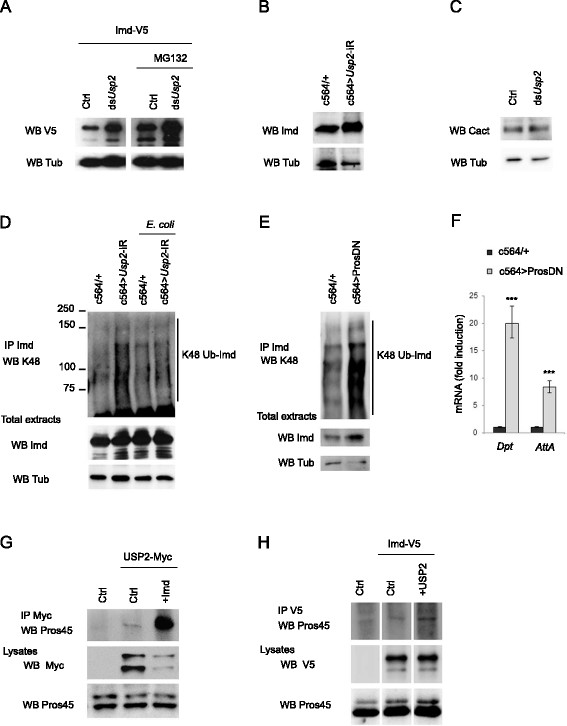
**Imd proteasomal degradation requires USP2. A**. S2 cells were transfected by Imd-V5 expressing construct and treated or not by *Usp2* silencing dsRNAs (ds*Usp2)*. Cells were incubated or not with MG132 at 20 μM. Cell lysates were immunoblotted with anti-V5 antibodies (WB V5). **B**. Protein extracts of c564/+ control or c564/Usp2-IR flies were immunoblotted with anti-Imd antibodies (WB Imd). **C**. S2 cells lysates treated or not with *dsUsp2* were immunoblotted with anti-Cact antibodies (DSHB) (WB Cact). **D**. Endogenous Imd was immunoprecipitated from extracts of c564/+ or c564/*Usp2*-IR flies infected or not by *E. coli*. Ubiquitinated forms of Imd were detected with anti-Ub^K48^ antibodies (IP Imd WB K48). Total extracts were immunoblotted with anti-Imd antibodies (WB Imd). **E**. Endogenous Imd was immunoprecipitated from extracts of c564/+ or c564/Pros26[[1]]; Prosbeta[[1]]/+ flies (c564 > ProsDN). Ubiquitinated forms of Imd were detected by anti-Ub^K48^ antibodies (IP Imd WB K48). **A**-**E**: Anti-tubulin immunoblots served as loading control (WB Tub). **F**. Quantitative analysis of *Dpt* and *AttA* mRNAs from c564 > ProsDN compared to c564/+ control flies. Histograms present the fold activation of each mRNA. Error bars indicate standard deviation between technical triplicates. Significant differences with control (*t*-test) p < 0.001 (***). **G**. S2 cells were cotransfected with pAc-USP2-Myc and either pAc-Imd or a control empty plasmid (Ctrl). USP2 was immunoprecipitated with anti-Myc antibodies and immunoblotted with anti Pros45 antibody (DHSB) (IP Myc WB Pros45). Cells lysates were immunoblotted with anti-Myc and anti Pros45 antibodies (WB Myc, WB Pros45). **H**. S2 cells were cotransfected with pAc-Imd-V5 and either pAc-Usp2 or a control empty plasmid (Ctrl). Imd was immunoprecipitated with anti-V5 antibodies and endogenous Pros45 was detected (IP V5 WB Pros45). Whole cells lysates were revealed with anti-V5 and anti Pros45 antibodies (WB V5 WB Pros45).

Our data indicate that Imd is permanently linked by Ub^K48^ putatively ensuring its turnover through proteasomal degradation. Indeed, inhibiting the proteasome in S2 cells with MG132 led to the accumulation of full-length and cleaved Imd when compared to untreated control cells (Figure 6A). Remarkably, silencing *Usp2* also provoked a clear accumulation of full length and cleaved Imd compared to control cells reaching similar amount as in MG132 treated cells (Figure 6A). Likewise, silencing of *Usp2* in the fat body resulted in the accumulation of endogenous Imd (Figure 6B). This is surprising because we have previously shown that USP2 promotes the hydrolysis of Ub^K48^ linked to Imd, a process which is expected to save Imd from proteasomal degradation. On the opposite, our results clearly indicate that *Usp2* silencing induces a blockade in the Imd degradation process thus suggesting that USP2 is required for Imd degradation. By contrast, the amount of the Toll pathway component Cactus was not modified by silencing *Usp2* in S2 cells (Figure 6C).

Since a proper immune response would require stable Imd, we investigated whether bacterial infection modifies the amount of Ub^K48^-linked and full length Imd. Strikingly, Ub^K48^-Imd accumulation seen in *Usp2*-silenced flies was strongly reduced in infected flies compared to uninfected flies indicating that the linkage of Ub^K48^ on Imd is actively prevented in response to immune challenge, thus allowing for Imd stabilisation (Figure 6D). To investigate whether accumulation of Imd protein could be sufficient to induce ectopic activation of the Imd pathway *in vivo*, we blocked proteasome function in flies expressing two conditional dominant-negative proteasome subunits (UAS-pros26^1ts^; UAS-prosβ2^1ts^) [[28]]. Flies kept at the restrictive temperature (30°C) for 72 hours accumulated Ub^K48^ –linked and full length Imd (Figure 6E) and concomitantly, they displayed significant activation of the *AttA* and *Dpt* expression (Figure 6F). Altogether, our results suggest an essential role of USP2 in both the hydrolysis of Ub^K48^ linked to Imd and the proteasomal degradation of Imd thus preventing its accumulation and subsequent constitutive activation of the Imd pathway.

### USP2 and Imd synergistically interact with the proteasome

To investigate how USP2 might ensure its regulatory function on Imd homeostasis, we realised a pull-down assay using S2 cell lysate and GST or GST-USP2 as baits to identify USP2-interacting proteins. Eluates were then analysed by a tandem mass spectrometry-based proteomic approach. Among other candidates specifically identified as associated with GST-USP2 (Additional file 3: Table S2), the proteasomal subunit 8 (Pros45) may trigger USP2 binding to the proteasomal machinery. Indeed, endogenous Pros45 co-immunoprecipitate with USP2-Myc (Figure 6G). Interestingly, a much higher amount of this proteasomal subunit was detected in the presence of overexpressed Imd (Figure 6G). Reciprocally, while Pros45 was hardly detectable in the purified product from immunoprecipitated Imd-V5, it was clearly observed when USP2 was overexpressed (Figure 6H). Thus, the Imd–USP2 complex apparently binds more efficiently to the proteasome than do Imd or USP2 alone. Taken together, our data suggest that USP2 and Imd synergistically bind to the proteasome and that USP2 contributes to Imd degradation at the proteasome level.

## Discussion

By using an RNAi screening approach, we demonstrate here the importance of three ubiquitin proteases, USP2, USP34 and USP36, in the regulation of the Imd immune signalling pathway. In addition, USP34 was identified as the only negative regulator of the Toll pathway in this screen. While this study and previous work [[17]] show a clear negative regulatory function for USP2 and USP36 on the Imd pathway in both un-infected and infected flies, USP34 can have both negative, (i.e., on *AttA, Drs* and *IM1*) and a positive (i.e., on *Dpt* and *Def*), regulatory functions on antimicrobial peptide genes in infected flies. Similar differential effect on various antimicrobial peptide genes induction has been observed with the POSH E3 ligase targeting TAK1 [[29]] while only a subset of Imd target genes (i.e., *Dpt* and *Dro* but not *AttA* and *CecA1*) are affected in a *faf* (*fat facets*) mutant, where *faf* encodes a deubiquitinating enzyme targeting Imd [[30]]. Our result suggest a complex requirement of USP34 putatively acting at different levels of these two pathways. In unchallenged conditions, USP34 may interfere with common negative regulatory mechanisms preventing ectopic transcriptional activation of NF-κB target genes while in challenged conditions, USP34 may target components that are differentially required in the two pathways.

**Figure 7:**
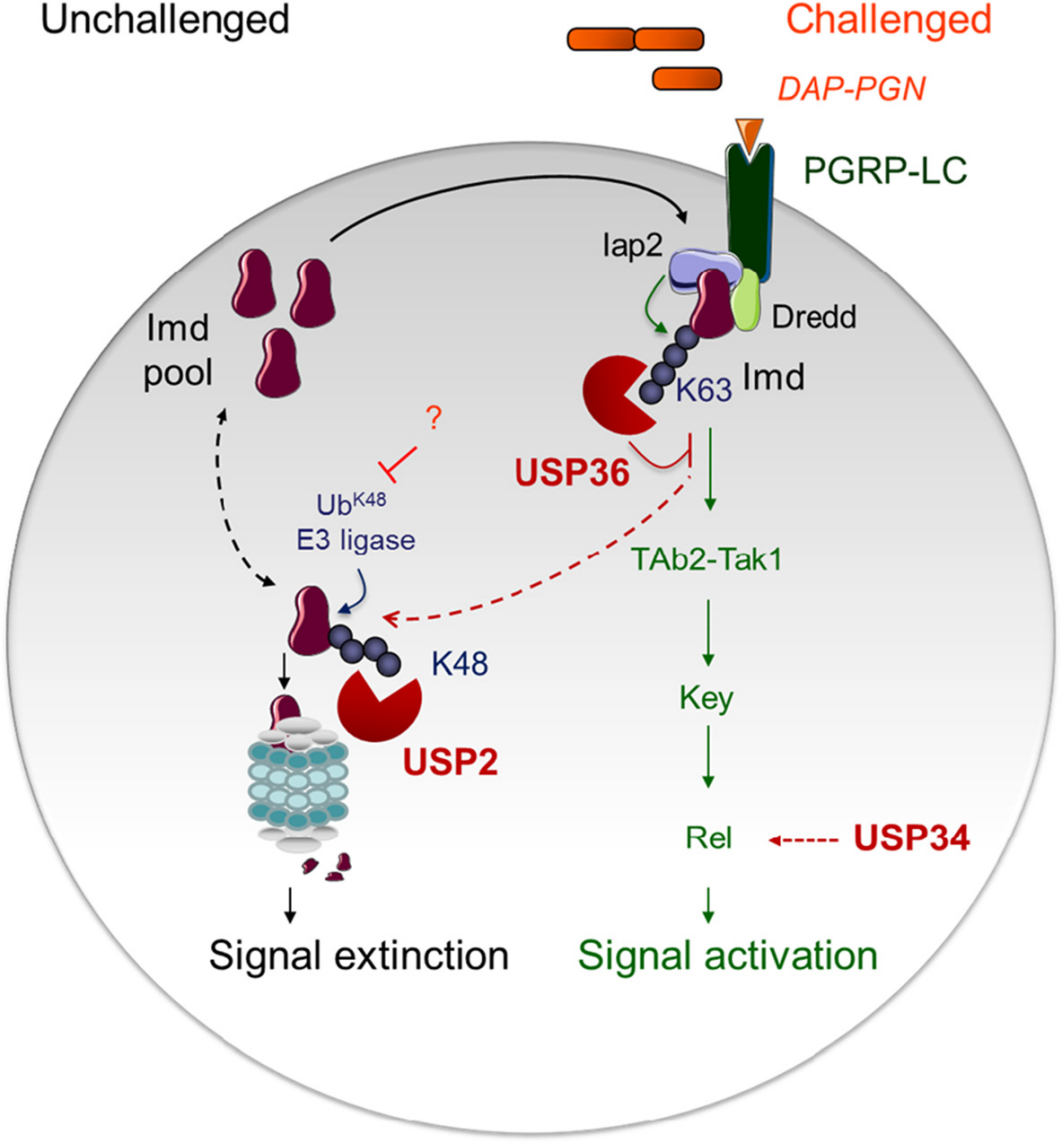
**(Summary illustration): Three USPs regulate Imd activation or stability and downstream signalling.** Three ubiquitin specific proteases regulate the Imd pathway, USP34 is putatively acting at the level of NF-κB like factors associated complexes (Rel in the case of Imd pathway) while USP2 and USP36 differentially targets the scaffolding molecule Imd by hydrolysing Ub^K48^ (this study) and Ub^K63^ [[17]] chains, respectively. Imd is subjected to a permanent turn-over via the linkage of Ub^K48^ ubiquitin chains and subsequent proteasomal degradation. This prevents Imd accumulation and constitutive signal activation in unchallenged conditions. The deubiquitinating enzyme USP2 is required for both Ub^K48^ hydrolysis and Imd degradation at the level of the proteasome. In response to an immune challenge, the activity of the E3 ligase would be prevented resulting in the observed Imd stabilisation and accumulation. It has been previously described that Imd activation in response to immune challenge results in its cleavage by Dredd and linkage of Ub^K63^ by Iap2 [[31]]. USP36 deubiquitinates Imd linked Ub^K63^ thus preventing inappropriate or excessive signal transduction and putatively promoting ubiquitin chain editing through the replacement of activating Ub^K63^ by Ub^K48^ degradative chains [[17]].

Focusing on the function of USP2, we found that it interacts with Imd and specifically promotes the hydrolysis of Ub^K48^ from the protein, thus displaying different substrate specificity than USP36 which was previously described to promote the hydrolysis of Ub^K63^ from Imd [[17]]. Whereas the linkage of Ub^K63^ on Imd activates signal transduction [[17],[31]], we show here that Ub^K48^ serves as a tag for Imd degradation by the proteasome. Thus, differential ubiquitination of Imd regulates either its activity or its stability and is tightly controlled by the complementary activity of USP36 and USP2 (Figure 7). Even though they target different ubiquitin chains and have different effects on Imd, both enzymes contribute to down-regulation of the Imd pathway.

Actually, it was first rather surprising that while USP2 promotes the cleavage of Ub^K48^ from Imd, its physiological function is not to rescue Imd from degradation but rather to promote its degradation by the proteasome. This was clearly demonstrated by using silencing experiments: in *Usp2*-silenced cells, or in flies expressing the *Usp2-IR* silencing transgene, Ub^K48^ and full-length Imd accumulated indicating a blockade of the Imd degradation process. At the molecular level, the simplest explanation for this result is that USP2 drives the Ub^K48^-Imd molecules to the proteasome and/or ensures the removal of Ub^K48^ from Imd at the level of the proteasome, then allowing for Imd degradation by the proteasome machinery (Figure 7). Indeed, the release of attached polyubiquitin chain is necessary to permit the entry of a specific substrate to the proteasomal cavity [[32],[33]]. Moreover, it has been recently illustrated that deubiquitination by the yeast enzyme Ubp3 –which interacts with the 26S proteasome subunit- can either save proteins from destruction or facilitate protein destruction by the 26S proteasome, depending on stress conditions and Ubp3 amount in the cell [[34]]. Our evidence that USP2 interacts with Pros45, the 26S subunit 8 of the proteasome, and that this interaction is significantly enhanced in the presence of Imd supports similar essential role of USP2 in Imd degradation at the level of the proteasome. Interestingly, artificial overproduction of USP2 by tissue-directed or heat-shocked-induced expression of the *Usp2* expressing transgene in non-infected flies, also leads to Imd accumulation (data not shown). Therefore, as shown for Ubp3, depending on its total amount, USP2 may either save a target protein from degradation by the proteasome or favour its degradation.

The accumulation of Imd in *Usp2*-silenced flies most probably contributes to the observed constitutive induction of Imd-dependent AMP gene expression. Consistent with this, blocking the proteasome by expressing dominant-negative proteasome subunits in transgenic flies similarly resulted in accumulation of full length Imd concomitantly with constitutive activation of the Imd pathway. The molecular mechanisms underlying ectopic activation of the Imd pathway, when Imd is overexpressed or inefficiently degraded, are not known. Notably, it should be assess in the future whether it is associated or not with increased Imd K63-ubiquitination. Actually, in the absence of immune challenge that would activate the E3 ligase Iap2, which is responsible for the linkage of K63 chains on Imd [[31]], Imd accumulation and self-aggregation may favour the self-assembly of activating complexes independently of the linkage of ubiquitin moieties. Interestingly, similar activation of *Dpt* expression was previously observed following proteasomal inhibition and was associated with Rel stabilisation [[35]]. Thus, proteasome activity, by acting on several components of the pathway (at least Imd and Rel), likely has an important function in damping down immune signalling in the absence of infection.

Remarkably, the accumulation of Ub^K48^ -Imd seen in *Usp2*-silenced flies mostly disappeared following an immune challenge. Subsequently, Imd is significantly stabilised in challenged flies compared to unchallenged flies. Since *Usp2* was silenced in these flies, this disappearance of Ub^K48^ -Imd cannot be a consequence of USP2 deubiquitinating activity. Rather, we suggest that infection inhibits the linkage of Ub^K48^ to Imd by an unidentified E3 ubiquitin ligase and/or activate another DUB that would cleave K48 ubiquitin chains linked to Imd.

The human homologs of USP2 and USP34 also play regulatory roles in NF-κB-dependent immune signalling. One study reported that USP34 is a regulator of T-cell receptor (TCR)-dependent lymphocyte activation where it acts as a down-regulator of NF-κB signalling [[36]]. Although its molecular target remains to be identified, USP34 functions downstream of the IKK complex in human cells, putatively on proteins that would be commonly required in these different NF-κB signalling pathways. The fact that USP34 is required in the two NF-κB immune signalling pathways Toll and Imd also argues in favour of USP34 targeting common regulatory mechanisms at the level of NF-κB transcription factors. This hypothesis is reinforced by the observation that in response to infection, USP34 differentially regulates AMP genes expression which may typically reflect differential activity at the level of NF-κB-containing complexes. Of note, however, co-silencing of the scaffolding proteins Imd or dMyd88 fully disrupted the PGRP-LC- or the Toll-dependent activation of immune signals observed in *Usp34*-silenced condition, respectively. This suggests that these upstream molecules are required to ensure a minimal activation of NF-κB-like factors that are then tightly regulated by USP34-dependent mechanisms that stay to be discovered in both *Drosophila* and mammalian models.

A set of different studies point to a regulatory function of the mammalian USP2a isoform on TNF-R1 dependent activation of the NF-κB pathway. First, silencing *Usp2a* contributes to TNFα-dependent hepatocyte survival in mice, presumably due to enhanced transcriptional activity of NF-κB [[37]]. Second, USP2a hydrolyses Ub^K63^ from RIP1 thus preventing NF-κB activation while promoting caspase-mediated cell death in response to TNFα stimulation in human cells [[38]]. Two other studies, by contrast, provide controversial results by describing a positive role for USP2a in TNF-R1 dependent NF-κB signalling [[39]] or in the regulation of TCR-induced NF-κB activation [[40]]. These discrepancies may be due to dose-dependent effects of USP2 on multiple targets.

## Conclusion

In addition to the NF-κB pathway, a huge and increasing number of proteins and pathways seems to be controlled by USP2, which is also deregulated in many cancers [[41]-[46]]. Our demonstration of USP2 involvement in the proteasomal degradation of Imd suggests a more extended function at the proteasome level than previously anticipated. It may highlight new mechanisms of action of USP2 on its targets and provide some molecular explanations for its tumorigenic function in humans in the future.

## Methods

### Cell culture and RNA silencing

*Drosophila* S2 cells were maintained in Schneider’s *Drosophila* medium supplemented with 10% heat-inactivated serum (FCS, Invitrogen). Gene inactivation was achieved by incubating 0.4 μg double strand RNA (dsRNA) for 48 h at 26°C with 1.2×10^5^ S2 cells cultured in 96-well microplates (adapted from [[47]]. DNA templates for dsRNA synthesis were generated by PCR (MEGAscript RNAi kit, Ambion) using the primers designed from Heidelberg Fly Array RNAi libraries (www.genomernai.org/). To monitor Imd and Toll pathways activation, we used the reporter constructs *pAttA-luc* [[48]] and *pDrs-luc* [[21]], respectively. We further constructed a normaliser *pAct-luc* (Promega). DNA transfection was performed 48 hours prior to luciferase detection (simultaneously with dsRNAs). To activate the Imd pathway, heat-killed *E. coli* (5×10^7^) were added 4 hours prior to dual detection of the two luciferases.

### Fly strains and infections

Flies were raised on standard culture medium at 25°C except if indicated. The insertion P[EPgy2]ash2EY03971 was obtained from BDSC. Transgenic lines were constructed in the P[UAST] vector [[23]]. The P[UAS-*Usp2*] transgene contains the *CG14619-RA* cDNA subcloned from SD02480 (DGRC). Heat-shock driven expression of *Usp2* or *Usp34* was achieved as described in [[17]]. For *in vivo* gene silencing, inverted repeats designed from the Heidelberg RNAi librarie (HFA, www.genomernai.org/) –that are similar as those used for S2 cell silencing- of *Usp2* and *Usp34* were cloned in the PWIZ vector [[49]]. Other silencing transgenes, P[UAS-*Imd-IR*] (#9253); P[UAS-*Usp2-IR*](#37930) and P[UAS-*Usp34-IR*] (#27517) were obtained from VDRC [[25]]. Importantly VDRC lines display different silencing sequences than our home-made constructs. For infection, fifty 3–5 days old males were pricked in the thorax with a thin needle that had been previously dipped in a concentrated overnight culture (OD_600_ #400) of *E. coli* or *M. luteus* (to measure activation of the Imd and Toll pathways respectively), or of *K. pneumonia* or *E. faecalis*, to measure flies survival.

### Real-time quantitative PCR (RT-qPCR) analysis

Total RNAs were extracted from adult flies using Absolutely RNA Miniprep kit from Stratagene. For real-time PCR analysis, cDNAs were synthesized with AffinityScript QPCR cDNA Synthesis Kit (Stratagene). An amount of cDNA equivalent to 0.01 μg of total RNA was subjected to 40 cycles of PCR amplification consisting of a 10s incubation at 95°C and 30s at 60°C. Output was monitored using SYBR Green core reagents and the Mx3000P instrument (Stratagene). All the results were normalized to the *rpl32* RNA level. The primer sequences were designed using PrimerQuest (http://eu.idtdna.com/Scitools/Applications/Primerquest/).

### *In vitro* deubiquitinating assays

Mutation in the *Usp2* coding sequence (resulting in the mutated protein USP2C540S) was introduced using QuickChange XL Site-directed Mutagenesis Kit (Stratagene). Wild type or mutated catalytic domains of USP2[AA 475 to 856] were coexpressed with Ub-β-gal fusion protein for 4 hours at 28°C in transformed *E. coli* XL1 Blue. Bacteria were lysed in 100 μL Laemmli solution. Samples were subjected to SDS-PAGE (6% gel) and western blotting with a rabbit anti β-Gal antibody (ROCKLAND).

### Immunoprecipitation and immunoblotting

Co-immunoprecipitation were performed following standard procedures in S2 cotransfected cells with 10 μg of Myc tagged full length USP2 construct in pAc/HisB vector (Invitrogen) (USP2-Myc) and 10 μg of V5-tagged Imd full length or truncated constructs. Pull Down assays were performed in S2 cells transfected with full length Imd-V5 construct and lysed after 48 h. The lysate was employed in a GST-USP2 *N*-ter domain [AA 1 to 531] or *C*-ter domain [AA 475 to 856] pull down assays. Pull downs were blotted with antibody against V5 to detect bound Imd-V5.

### Proteomic analysis (supporting data deposited in a database)

Protein digestion and nano-liquid chromatography (LC)–MS/MS analyses were performed as described in [[50]]. Only proteins identified specifically in the USP2 sample and not in the control one, with a minimum of 6 peptides and specific spectral counts above 6, were retained (indicated in green in Additional file 3: Table S2). The mass spectrometry proteomics data have been deposited to the ProteomeXchange Consortium (http://www.proteomexchange.org) via the PRIDE partner repository [[51]] with the data set identifier PXD000881 and doi:10.6019/ PXD000881.

## Competing interests

The author declare that they have no competing interests.

## Authors’ contribution

EE, MM, and DT performed S2 cells screens, flies assays, RT-qPCR experiments and biochemical analyses. PV performed RT-qPCR experiments. YC did mass spectrometry analysis. EE, DT and ET help in the design of the experiments and in manuscript drafting. MOF designed the experiments, supervised the study and wrote the manuscript. All authors read and approved the final manuscript.

## Additional files

## Supplementary Material

Additional file 1: Table S1. Ubiquitin proteases screened on Imd and Toll pathways. List of Usps and corresponding CG numbers screened on Imd and Toll pathway in S2 cells with indication of human closest homolog gene(s). cDNA templates and primers used for dsRNA synthesis are indicated. All primers were designed from (http://www.genomernai.org/). Of note, the USP encoding gene CG8232 was not included in this study [[7],[19]]. The asterix (*) indicates the *Usp* genes not screened in [[7]]. The double asterix (**) indicates an alternative nomenclature used in [[7]]. Click here for file

Additional file 2: **Figure S1.** Analysis of *Usp36*, *Usp34* and *Usp2* gene extinction by RT-qPCR in S2 cells treated for 48 h with the indicated dsRNAs ds*GeneName*). **Figure S2.** Activation of *Drs* promoter was monitored through the p*Drs*-luc reporter (normalised to p*Act*-luc) in transfected cells expressing pAc-Spz and treated for 48 h with the indicated dsRNAs. NI: no inducer. **Figure S3.** Analysis of *Usp2* and *Usp34* expression by RT-qPCR in HspGal4/+;UAS-Usp2/+ (Hsp>Usp2) or HspGal4/+; P{EPgy2}ash2EY03971 (Hsp>Usp34) compared to Hsp/+ flies at indicated time points after heat shock. **Figure S4.** Analysis of *Usp2* (A), *Usp34* (B) or *Dpt* (C,D) expression by RT-qPCR in total flies (A,C,D) or in dissected guts (B). Indicated silencing transgenes were induced by heat shock (HS-Gal4) (A) or in the gut (NP1-Gal4) (B) or in the fat body (c564-Gal4) (C,D). **Figure S5.***Dpt*, *AttA* or *DefC* expression in c564-Gal4/Usp2-IR (#5 M) (A) or c564-Gal4/Usp34-IR (#1 M) (B,C) compared to c564-Gal4/+ flies. A,C: Flies were infected by *E. coli* by a septic injury (100% of activation fixed at 3 h post-infection). **Figure S6.** Analysis of *Usp2* (A) or *Usp34* (B) expression in flies by RT-qPCR at indicated time points following infection with *E. coli* or *M. luteus*. NI: not infected flies. Slight differences in gene expression were considered not biologically significant (below 1.6 fold). **Figure S7.** USP2 preferentially interacts with Imd-*N*ter. A. Representation of Imd full length (Imd-FL) and truncated constructs used in GST pull down assays. CD: catalytic domain in USP2, C540 catalytic cysteine, DD: death domain in Imd. B. S2 cells were transfected with pAc-USP2-Myc and lysed after 48 h. Cells lysates were pre-cleared and subjected to GST pull down using the indicated GST fusion proteins: Imd FL, Imd-*N*ter or the Imd-*C*ter. USP2-Myc was detected by western blot with anti-Myc antibodies. Click here for file

Additional file 3: Table S2. List of USP2 interacting proteins identified by a Mass spectrometric analysis. Proteins identified specifically in the USP2 sample and not in the GST control one with a minimum of 6 peptides and specific spectral counts above 6 are indicated. The full mass spectrometry proteomics data have been deposited to the ProteomeXchange Consortium (http://www.proteomexchange.org) via the PRIDE partner repository [[51]] with the data set identifier PXD000881 and doi:10.6019/ PXD000881. Click here for file
