# Supplementary material for: Identifying USPs regulating immune signals in Drosophila: USP2 deubiquitinates Imd and promotes its degradation by interacting with the proteasome
Source: Cell Commun Signal. 2014 Jul 16;12:41. doi: 10.1186/s12964-014-0041-2 (PMC4140012; doi:10.1186/s12964-014-0041-2)
Supplement: Additional file 1: Table S1. — Ubiquitin proteases screened on Imd and Toll pathways. List of Usps and corresponding CG numbers screened on Imd and Toll pathway in S2 cells with indication of human closest homolog gene(s). cDNA templates and primers used for dsRNA synthesis are indicated. All primers were designed from (http://www.genomernai.org/). Of note, the USP encoding gene CG8232 was not included in this study [[7],[19]]. The asterix (*) indicates the Usp genes not screened in [[7]]. The double asterix (**) indicates an alternative nomenclature used in [[7]]. [file s12964-014-0041-2-S1.doc]

**Supplemental Table 1: USPs encoding genes screened on Imd and Toll pathways**

| **Human closest homologs** | **Drosophila gene** | **CG number** | **Template** | **Forward and Reverse primer (without T7 sequences)** | **amplicon ID** |
| --- | --- | --- | --- | --- | --- |
| USP1 | Usp1 | CG15817 | LD22910 | TACATAGCCAACCCGGATG  CACAATGCCTTCGAAGTCC | HFA14155 |
| USP2 | Usp2 | CG14619 | SD04280 | CCAGCAGACCAAGGACACTC  GAGCTGCTGCCGGATCCGCC | HFA20632 |
| USP5 | Usp5 | CG12082 | Genomic DNA | CCAGATTGGTGGCCTTGTC  CTGGCATGCGCAATCTG | HFA08244 |
| USP7/HAUSP | Usp7 | CG1490 | LD41613 | GTCAGCGTCAACGGGAG  CGACGACGGCAGTGTTT | HFA18909 |
| USP8 | UbpY/Usp8 | CG5798 | Genomic DNA | CAGAACGATGAGCAGTTGGA  CCTCGGCTGCGGTTTCATCG | HFA15876 |
| USP10 | Usp10* | CG32479 | LD28815 | GGACGGATGCGCGAACGAGG  CTGGTGCCAGATCTGGGTCA | HFA08282 |
| USP12/USP46 | Usp12-46 | CG7023 | RE52890 | TACTGGCCGAACGGAATG  TTCTGCGCCTCCTGATAA | HFA16175 |
| USP14 | Usp14 | CG5384 | Genomic DNA | TAGTTTCTCCAAACGTCTGG  CTTGGTGAGCCGACTTCC | HFA02853 |
| USP15/USP31 | Usp15,  Usp31** | CG30421 | GH27809 | TACCGAAATGATCGACATGG  CCAGCCCGAGTCCAAGTCCA | HFA04426 |
| USP16/USP45 | Usp16, Usp45** | CG4165 | LD34905 | GTATTCCGTGGCTTTCTTGT  GGTCTGGCCGTTGGTCTGTT | HFA18352 |
| USP30 | Usp30 | CG3016 | LD41827 | GTTGCGGCGGCAGATTTAGC  CCCTCCAGTGCCATGCTCAC | HFA18265 |
| USP32 | Usp32 | CG8334 | Genomic DNA | GCTGTTTAATACCCAACCACT  AGACCTCAAAGTAGATATAATTCT | HFA10979 |
| USP33/USP20 | Usp33, Usp20** | CG8494 | LD43649 | TCCGCTGGAGGGCTTCGACA  TCCGCCACCGAAAGTCCGGT | HFA07196 |
| USP34 | Usp34 | CG5794 | Genomic DNA | GCCATGCGACTGATCTCT  CCGCACGATGGTGATCTT | HFA15875 |
| USP38/USP35 | Usp38 | CG8830 | LD36231 | CATCGATAGGTGGTGGAAAG  TGCTTATCGAGTGCAATTCA | HFA07281 |
| USP39 | Usp39 | CG7288 | LD38070 | CATCAAAGCGAACGACTAC  GGTAGATCGCATGTGAGGT | HFA20032 |
| CYLD | Cyld | CG5603 | Genomic DNA | TCAATTATGTCTGTGACATCAAG  TCGGGGCAAGATTCTTATTT | HFA02870 |
| USP9 | fat facets* | CG1945 | LD22582 | TGACATCCTGAAGCAGAACT  TAACCATGTATACGAACGGTC | HFA16942 |
| USP22 /USP51 | Not* | CG4166 | LD43147 | CGCAGGCTGAACTGTTTGGT  TCTATTCCGGCTCCCGTTCG | HFA11378 |
| USP36 /USP42 | Scny/dUsp36/Usp36 | CG5505 | LD40339 | GTGCGTTTGACCAACGGACA  AGTGGCGTTTTGACGGGAGT | HFA10510 |
| USP47 | Ubp64E/  E(var)3-64E | CG5486 | LD26783 | ACCAGAGTGTACGTATCGC  TGGGTGTTGGCCATGTTCT | HFA11313 |
