# Supplementary material for: Identifying USPs regulating immune signals in Drosophila: USP2 deubiquitinates Imd and promotes its degradation by interacting with the proteasome
Source: Cell Commun Signal. 2014 Jul 16;12:41. doi: 10.1186/s12964-014-0041-2 (PMC4140012; doi:10.1186/s12964-014-0041-2)
Supplement: Additional file 2: — Figure S1. Analysis of Usp36, Usp34 and Usp2 gene extinction by RT-qPCR in S2 cells treated for 48 h with the indicated dsRNAs dsGeneName). Figure S2. Activation of Drs promoter was monitored through the pDrs-luc reporter (normalised to pAct-luc) in transfected cells expressing pAc-Spz and treated for 48 h with the indicated dsRNAs. NI: no inducer. Figure S3. Analysis of Usp2 and Usp34 expression by RT-qPCR in HspGal4/+;UAS-Usp2/+ (Hsp>Usp2) or HspGal4/+; P{EPgy2}ash2EY03971 (Hsp>Usp34) compared to Hsp/+ flies at indicated time points after heat shock. Figure S4. Analysis of Usp2 (A), Usp34 (B) or Dpt (C,D) expression by RT-qPCR in total flies (A,C,D) or in dissected guts (B). Indicated silencing transgenes were induced by heat shock (HS-Gal4) (A) or in the gut (NP1-Gal4) (B) or in the fat body (c564-Gal4) (C,D). Figure S5.Dpt, AttA or DefC expression in c564-Gal4/Usp2-IR (#5 M) (A) or c564-Gal4/Usp34-IR (#1 M) (B,C) compared to c564-Gal4/+ flies. A,C: Flies were infected by E. coli by a septic injury (100% of activation fixed at 3 h post-infection). Figure S6. Analysis of Usp2 (A) or Usp34 (B) expression in flies by RT-qPCR at indicated time points following infection with E. coli or M. luteus. NI: not infected flies. Slight differences in gene expression were considered not biologically significant (below 1.6 fold). Figure S7. USP2 preferentially interacts with Imd-Nter. A. Representation of Imd full length (Imd-FL) and truncated constructs used in GST pull down assays. CD: catalytic domain in USP2, C540 catalytic cysteine, DD: death domain in Imd. B. S2 cells were transfected with pAc-USP2-Myc and lysed after 48 h. Cells lysates were pre-cleared and subjected to GST pull down using the indicated GST fusion proteins: Imd FL, Imd-Nter or the Imd-Cter. USP2-Myc was detected by western blot with anti-Myc antibodies. [file s12964-014-0041-2-S2.pdf]

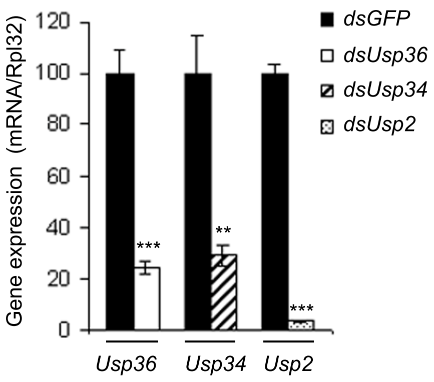

Suppl Figure 1

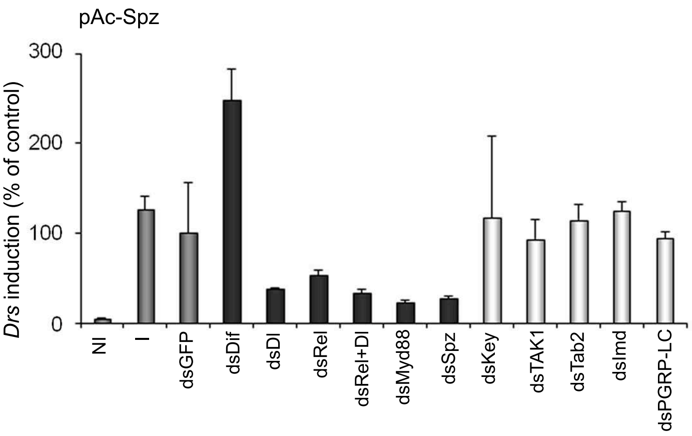

Suppl Figure 2

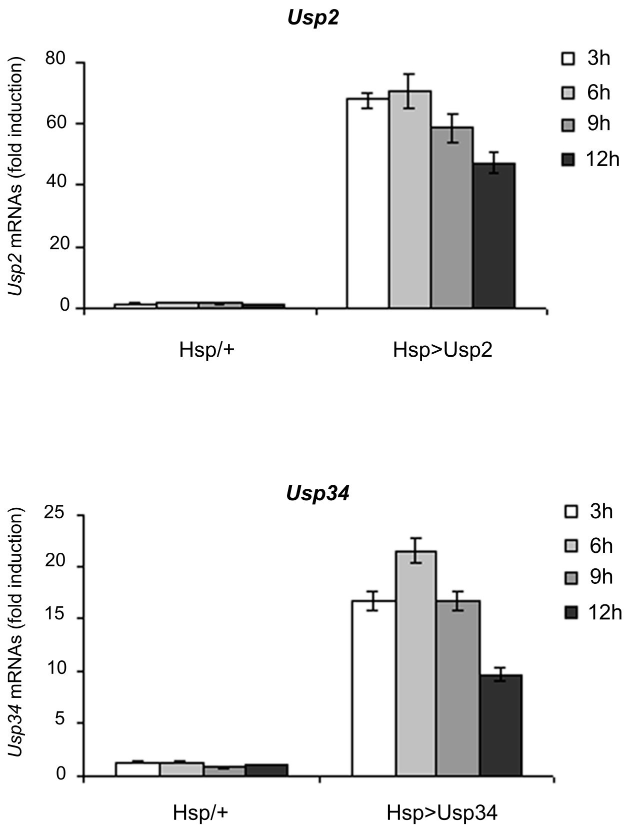

Suppl Figure 3

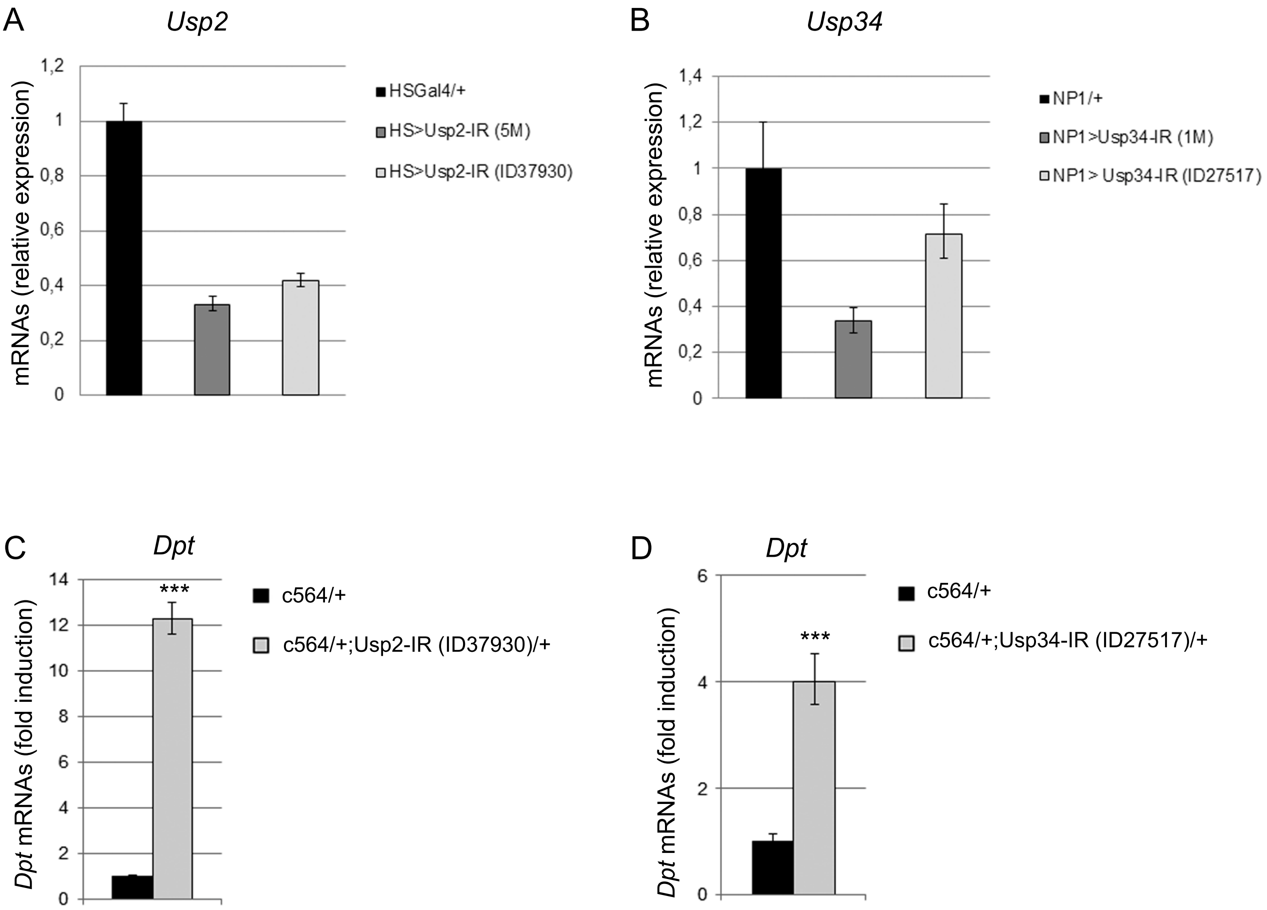

Suppl Figure 4

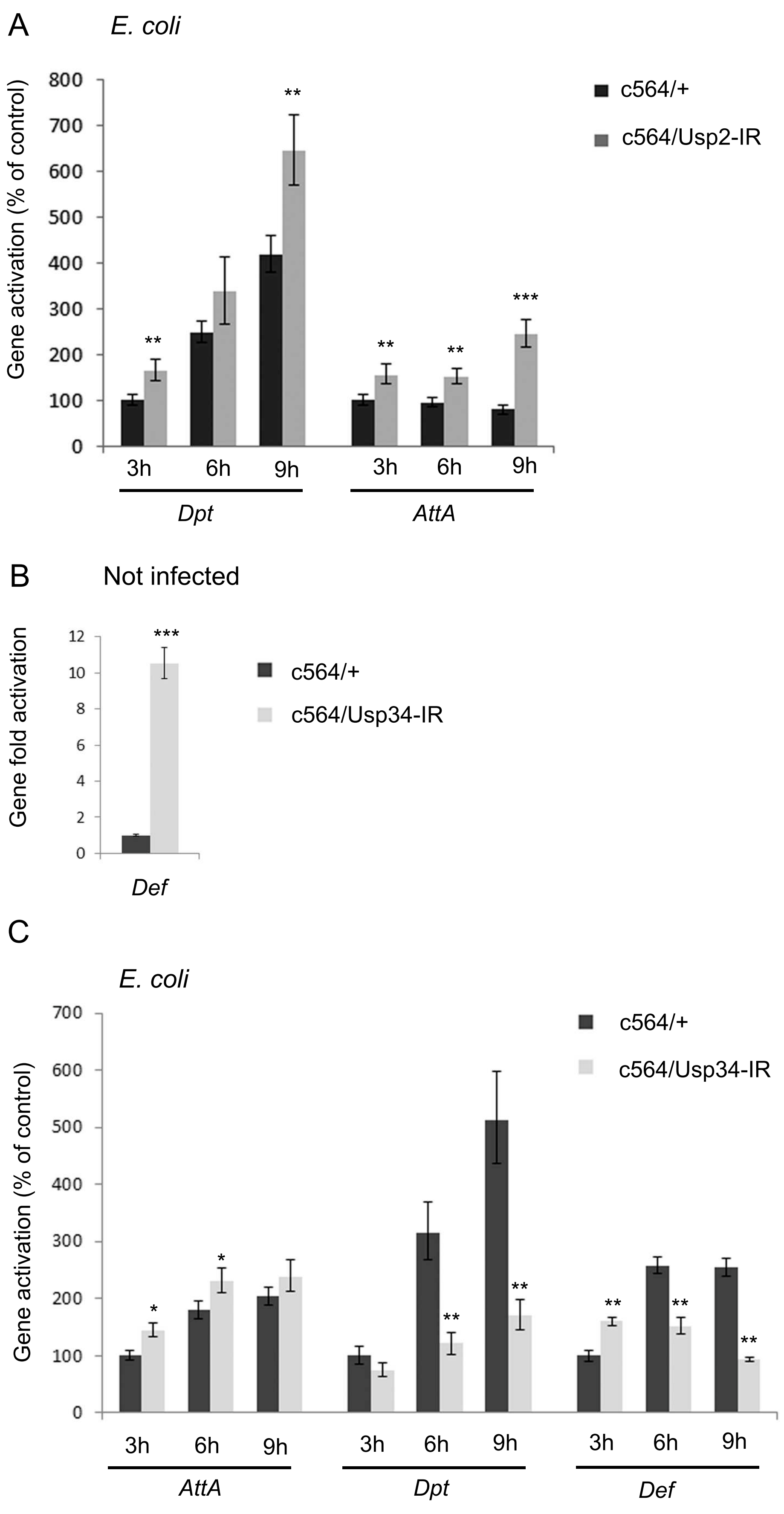

Suppl Figure 5

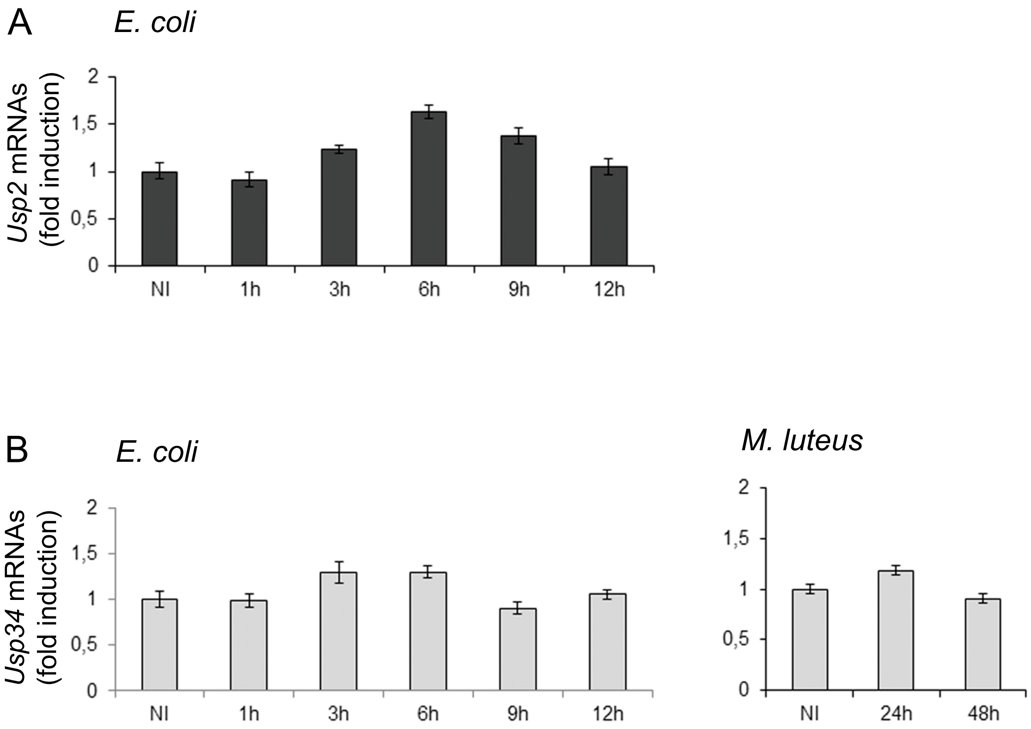

Suppl Figure 6

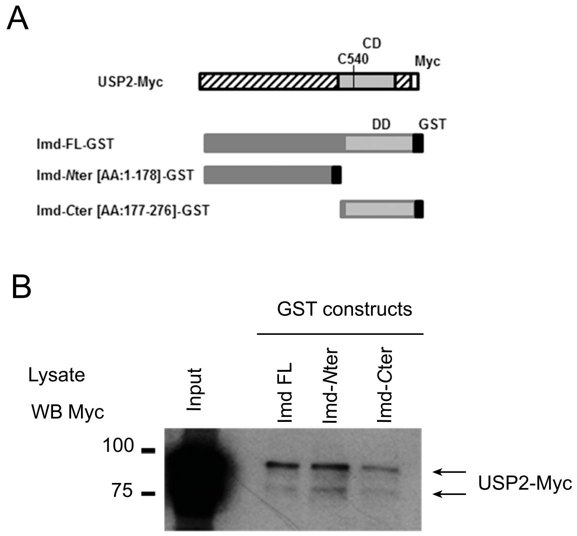

Suppl Figure 7
