# Supplementary material for: Identifying USPs regulating immune signals in Drosophila: USP2 deubiquitinates Imd and promotes its degradation by interacting with the proteasome
Source: Cell Commun Signal. 2014 Jul 16;12:41. doi: 10.1186/s12964-014-0041-2 (PMC4140012; doi:10.1186/s12964-014-0041-2)
Supplement: Additional file 3: Table S2. — List of USP2 interacting proteins identified by a Mass spectrometric analysis. Proteins identified specifically in the USP2 sample and not in the GST control one with a minimum of 6 peptides and specific spectral counts above 6 are indicated. The full mass spectrometry proteomics data have been deposited to the ProteomeXchange Consortium (http://www.proteomexchange.org) via the PRIDE partner repository [[51]] with the data set identifier PXD000881 and doi:10.6019/ PXD000881. [file s12964-014-0041-2-S3.pdf]

Accession	Protein name	SC USP2 / SC GST	Pep	SC	SSC
Q9VR54_DROME	Q9VR54_DROME Ubiquitin carboxyl-terminal hydrolase OS=Drosophila melanogaster GN=CG14619 PE=2 SV=2	220,5	48	441	441
Q9W0S7_DROME	Q9W0S7_DROME LD20211p OS=Drosophila melanogaster GN=Tudor-SN PE=1 SV=1	USP2 only	12	12	12
A1ZBE9_DROME	A1ZBE9_DROME CG15100 OS=Drosophila melanogaster GN=CG15100 PE=3 SV=1	USP2 only	12	12	12
Q9V434_DROME	Q9V434_DROME Asparaginyl-tRNA synthetase, isoform A OS=Drosophila melanogaster GN=Aats-asn PE=2 SV=1	USP2 only	11	13	13
Q8IPE8_DROME	Q8IPE8_DROME Mitochondrial trifunctional protein alpha subunit, isoform B OS=Drosophila melanogaster GN=Mtpalpha PE=3 SV=1	USP2 only	11	11	11
Q9VGH5_DROME	Q9VGH5_DROME AT27789p OS=Drosophila melanogaster GN=glo PE=2 SV=1	USP2 only	9	10	10
PERQ1_DROME	PERQ1_DROME PERQ amino acid-rich with GYF domain-containing protein CG11148 OS=Drosophila melanogaster GN=CG11148 PE=1 SV=2	USP2 only	8	8	8
DCTN1_DROME	DCTN1_DROME Dynactin subunit 1 OS=Drosophila melanogaster GN=Gl PE=1 SV=2	USP2 only	8	8	8
RRP1_DROME	RRP1_DROME Recombination repair protein 1 OS=Drosophila melanogaster GN=Rrp1 PE=1 SV=2	USP2 only	7	8	8
RL7_DROME	RL7_DROME 60S ribosomal protein L7 OS=Drosophila melanogaster GN=RpL7 PE=1 SV=2	USP2 only	7	8	8
PRS8_DROME	PRS8_DROME 26S protease regulatory subunit 8 OS=Drosophila melanogaster GN=Pros45 PE=1 SV=2	USP2 only	7	7	7
Y2138_DROME	Y2138_DROME Formin-like protein CG32138 OS=Drosophila melanogaster GN=CG32138 PE=1 SV=3	USP2 only	6	6	6
Q9VZI1_DROME	Q9VZI1_DROME Calponin-like protein Chd64 OS=Drosophila melanogaster GN=Chd64 PE=2 SV=2	USP2 only	6	6	6
Q9VPU7_DROME	Q9VPU7_DROME Arouser, isoform C OS=Drosophila melanogaster GN=aru PE=2 SV=1	USP2 only	6	6	6
Q9VBU7_DROME	Q9VBU7_DROME Nup358 OS=Drosophila melanogaster GN=Nup358 PE=4 SV=2	USP2 only	6	6	6
Q9VAW3_DROME	Q9VAW3_DROME GH12731p OS=Drosophila melanogaster GN=Gfat2 PE=2 SV=1	USP2 only	6	6	6
EIF3B_DROME	EIF3B_DROME Eukaryotic translation initiation factor 3 subunit B OS=Drosophila melanogaster GN=eIF3-S9 PE=1 SV=1	USP2 only	6	6	6
COPG_DROME	COPG_DROME Coatomer subunit gamma OS=Drosophila melanogaster GN=gammaCop PE=2 SV=1	USP2 only	6	6	6

Pep number of peptides
SC spectral counting
SSC specific spectral counting

(green) only identified in USP2 pull-down (Pep, SC and SSC > 5)
